# CRISPR-Mediated Triple Knockout of SLAMF1, SLAMF5 and SLAMF6 Supports Positive Signaling Roles in NKT Cell Development

**DOI:** 10.1371/journal.pone.0156072

**Published:** 2016-06-03

**Authors:** Bonnie Huang, Julio Gomez-Rodriguez, Silvia Preite, Lisa J. Garrett, Ursula L. Harper, Pamela L. Schwartzberg

**Affiliations:** 1 Genetic Disease Research Branch, National Human Genome Research Institute, Bethesda, Maryland, United States of America; 2 Embryonic Stem Cell and Transgenic Mouse Core, National Human Genome Research Institute, Bethesda, Maryland, United States of America; 3 Genomics Core, National Human Genome Research Institute, National Human Genome Research Institute, Bethesda, Maryland, United States of America; Jackson Laboratory, UNITED STATES

## Abstract

The SLAM family receptors contribute to diverse aspects of lymphocyte biology and signal via the small adaptor molecule SAP. Mutations affecting SAP lead to X-linked lymphoproliferative syndrome Type 1, a severe immunodysregulation characterized by fulminant mononucleosis, dysgammaglobulinemia, and lymphoproliferation/lymphomas. Patients and mice having mutations affecting SAP also lack germinal centers due to a defect in T:B cell interactions and are devoid of invariant NKT (iNKT) cells. However, which and how SLAM family members contribute to these phenotypes remains uncertain. Three SLAM family members: SLAMF1, SLAMF5 and SLAMF6, are highly expressed on T follicular helper cells and germinal center B cells. SLAMF1 and SLAMF6 are also implicated in iNKT development. Although individual receptor knockout mice have limited iNKT and germinal center phenotypes compared to SAP knockout mice, the generation of multi-receptor knockout mice has been challenging, due to the genomic linkage of the genes encoding SLAM family members. Here, we used Cas9/CRISPR-based mutagenesis to generate mutations simultaneously in *Slamf1*, *Slamf5* and *Slamf6*. Genetic disruption of all three receptors in triple-knockout mice (TKO) did not grossly affect conventional T or B cell development and led to mild defects in germinal center formation post-immunization. However, the TKO worsened defects in iNKT cells development seen in SLAMF6 single gene-targeted mice, supporting data on positive signaling and potential redundancy between these receptors.

## Introduction

The SLAM family of costimulatory receptors is widely expressed in the hematopoietic system, and has diverse functions in lymphocyte development and function, including roles in regulating T lymphocyte adhesion, cytotoxicity, cytokine production and survival. The importance of this receptor family is highlighted by the human genetic immunodeficiency X-linked lymphoproliferative disease Type 1 (XLP1), which is caused by mutations affecting *Sh2d1a*, which encodes SAP, a small adaptor protein that regulates signaling from SLAM family members [1]. Patients who suffer from XLP1 have a severe immunodysregulation characterized by an inability to clear Epstein-Barr virus infections leading to fatal infectious mononucleosis, dysgammaglobulinemia, and lymphoproliferation that can progress to lymphomas [2]. Bone marrow transplantation is currently the main treatment for this severe and often fatal disease [3].

Studies of SAP-deficient (*Sh2d1a*^-/-^) mouse models have provided insight into this disease and revealed two additional phenotypes that have been confirmed in XLP1 patients: a lack of long-term humoral immunity associated with defective T cell help for germinal center (GC) formation [4], and the absence of invariant natural killer T (iNKT) cells [5–7]. Intravital imaging studies of T cells in response to immunization further revealed that SAP is critical for long lasting T:B cell interactions required for germinal center formation and the generation of follicular T helper cells in the germinal center [8]. These observation have led to the speculation that SAP deficiency specifically affects lymphocyte:lymphocyte interactions [9]. Other phenotypes associated with SAP-deficiency include an inability to kill EBV-infected B cells and other B cell targets [10–12] and decreased T lymphocyte cell death [13].

Understanding the contribution of the SLAM family members may help our understanding of XLP1, as well as the basic immunological processes disturbed in this disease. Six SLAM family members, including SLAMF1 (SLAM), SLAMF3 (Ly9), SLAMF4 (2B4), SLAMF5 (CD84), SLAMF6 (Ly108/NTB-A), and SLAMF7 (CRACC), have been found to signal through SAP, which is most abundantly expressed in T lymphocytes and NK cells [1]. However, mutations affecting these SLAM family members in mice lead to relatively mild phenotypes that do not recapitulate the phenotypes associated with SAP-deficiency, perhaps reflecting the expression of multiple SLAM family members on different lymphocyte populations. Thus, the relative contribution of SLAM family members to these phenotypes has remained difficult to elucidate.

This understanding has been further complicated by observations that the SLAM family receptors can signal in both positive and inhibitory manners. SAP (and a related adaptor EAT2 in NK cells) have been shown to signal in T and NK cells via recruitment of Src family kinases and other signaling molecules to facilitate positive signaling [1]. However, other data demonstrate that SLAM family members act as inhibitory receptors in the absence of SAP, recruiting the SHP-1 phosphatase and other inhibitory molecules to SLAM family members, including SLAMF1 (SLAM), SLAMF6 (Ly108/NTB-A), SLAMF4 (2B4) and SLAMF7 (CRACC) [10, 14–17]. Thus, SLAM family members have both positive and negative signaling depending on the presence or absence of SAP, and the severe phenotypes of XLP1 and SAP-deficient mice may result from negative signals from SLAM family member in the absence of SAP. These observations have further clouded interpretation of the functions of SLAM family members and their relative contribution to phenotypes associated with SAP-deficiency.

One such phenotype that has been of much interest is the ability of SAP to regulate GC formation. T follicular helper cells are the critical subset of CD4 cells that provide signals to B cells for germinal center formation [18]. Evaluation of SLAM family members demonstrate that both Tfh and GC B cells express high levels of three SLAM family members: SLAM (SLAMF1), CD84 (SLAMF5) and Ly108 (SLAMF6) [19, 20], all of which are homophilic. GC responses have been evaluated in mice in which each of these receptors has been mutated alone. Although SLAM affects T cell IL-4 expression both in vivo in response to viral infection [21] and in vitro [22, 23], SLAM-deficient mice do not show defects in GC formation [21]. Mice harboring a mutation of *Slamf5* show mild variable phenotypes in GC responses to NP-ova immunization, but not to sheep red blood cells [19, 24], nor viral infection [15]. However, both Ly108 and CD84 can mediate T cell adhesion in vitro, and in vitro conjugation assays suggest they may compensate for each other [19]. While mutations affecting *Slamf6* also show no phenotypes in GC formation, remarkably, mutation of *Slamf6* rescues defects in GC formation [15] and CD8 cytotoxicity directed against B cells [10] seen in the absence of SAP, suggesting that the phenotypes of SAP deficiency may result in large part due to negative signaling from this SLAM family member. Mutation of *Slamf6* also rescues development of iNKT cells in *Slamf6*^-/-^*Sh2d1a*^-/-^ mice, again supporting a major effect of negative signaling in the absence of SAP [15]. Nonetheless, *Slamf6*^-/-^ mice also have mild reductions of iNKT cells, supporting positive signaling from SLAM family members in this pathway. Mixed bone marrow chimeras further suggest that both SLAM and Ly108 positively contribute to iNKT development [25]. Thus, Ly108 is likely to signal via both positive and negative pathways affecting iNKT cell development.

A better understanding of signaling from SLAM family members and their potential functional redundancy may be facilitated by evaluation of mice carrying mutations in multiple SLAM family members. The generation of such mice, however, has been hampered by the fact that the SLAM family members are encoded in a gene cluster spanning ~385 kb on mouse and human chromosome 1. Since these genes are tightly linked, it is not possible to generate mice with multiple mutations by conventional breeding of mice carrying mutations of individual SLAM family members. Recently, the use of Cas9/CRISPR-mediated mutagenesis via injection into fertilized mouse eggs has been shown to be capable of rapidly generating mice with mutations in up to five genes in a single step [26]. The development of this technology now permits the generation of mice carry multiple mutations in adjacent genes.

In this work, we have used CRISPR-mediated mutagenesis to introduce mutations into the genes encoding three SLAM family members, SLAM (*Slamf1*), CD84 (*Slamf5*) and Ly108 (*Slamf6*) that are highly expressed on Tfh and GC B cells and are implicated in both positive and negative pathways involved in Tfh cell differentiation and function. Two of these family members, SLAM and Ly108 are also implicated in the generation of iNKT cells. We find that mutation of these three family members did not affect the development of conventional T and B cells, and resulted in variable decreases in GC development in response to immunization. However, mutation of these genes led to more profound defects in iNKT cell development than mutations affecting Ly108, providing further support for positive signaling by SLAM family members.

## Materials and Methods

### Mice

*Sh2d1a*^-/-^ [27] and *Slamf6*^-/-^ [28] mice have been previously reported. Animal husbandry and experiments were performed in accordance with approved protocols by the National Human Genome Research Institute’s Animal Use and Care Committee, National Institutes of Health, Animal Welfare Assurance number A-4149-01.

### Generation of Slamf156 mutant mice

For all three SLAMF genes, early coding exons were targeted: exon 2 for *Slamf1* and *Slamf6*, and exon 3 for *Slamf5*. Guide sequences with specificity scores >50 were chosen using crispr.mit.edu. Complementary oligos were designed for each sgRNA construct, to clone into the T7 sgRNA construct (Transposagen) for in vitro transcription. Each oligo contained the 20 nucleotide guide sequence, preceded by four nucleotide overhangs that were compatible with the BsaI digested site of the vector (S1 Table). If the guide started with one or two G’s, those bases were not included in the oligo sequence, because the 5’ overhang already includes GG. The vector was digested with BsaI for 2 h at 37C, and then gel-purified. Paired oligos were annealed (95C for 5 min, 65C for 5 min, room temperature for 1 h), and ligated into the BsaI-digested vector. Constructs were sequenced to confirm guide insertion, and then linearized with DraI. The constructs were used for *in vitro* transcription with the MEGAshortscript Kit (Ambion), and mRNA was purified using the MEGAclear Kit (Ambion), both according to manufacturer instructions. Donor oligos for injection 1 were ordered as Ultramers from IDT and used directly. Pronuclear injections of mice were performed by methods as described in Behringer et al. [29]. Fertilized eggs were collected from super ovulated C57BL/6J female mice (Jackson Laboratories) approximately 9 hours after mating to C57BL/6N male mice (Jackson Laboratories). The male pronucleus was injected at a continuous flow with approximately 2 picolitres of injection mix: *S*. *pyogenes* Cas9 mRNA (Trilink), sgRNA mRNA, and oligo donor (only for injection 1), diluted in 10 mM Tris, 0.25mM EDTA (pH 7.5). Concentrations for each injection session are provided in S1 Table. The injected eggs were surgically transferred to pseudopregnant CB6/F1 (Jackson Laboratories) recipient females. Founders were crossed to B6 mice, and the heterozygous F1 were crossed with each other to obtain homozygous F2 knockouts.

### Fluorescent PCR genotyping

Tail genomic DNA was isolated using the Qiagen DNEasy-96 kit, and diluted 5-fold with water. Fluorescent PCR amplification and analysis were performed as previously described [30]. Fluorescent PCR and other genotyping primers are listed in S2 Table.

### Antibodies, iNKT tetramer, and flow cytometry

Flow cytometry reagents used were: TCRb (H57-597, eBioscience), CD4 (RM4-5, eBioscience), CD8a (53–6.7, eBioscience), CD21 (8D9, eBioscience), CD23 (B3B4, eBioscience), CD44 (IM7, eBioscience), NK1.1 (PK136, eBioscience), CD1d tetramer (PBS57, NIH Tetramer Core Facility), 2B4 (2B4, BD Biosciences), Ly9 (Ly9ab3, Biolegend), B220 (RA3-6B2, eBioscience), CD19 (1D3, eBioscience), Fas (15A7, eBioscience), GL-7 (GL-7, eBioscience), PD-1 (RMP-130, Biolegend), CXCR5 (2G8, BD Biosciences), biotin goat anti-rat (cat# 112-067-003, Jackson Immunoresearch), fluorophore-conjugated SAv (eBioscience). CXCR5 staining was performed as previously described [31]. Dead cells were excluded by staining with LIVE/DEAD Fixable Aqua Dead Cell Stain (Thermo).

### In vitro culture and intracellular cytokine staining

Naïve CD4 T cells (CD4^+^CD25^-^CD62L^hi^CD44^lo^) were sorted and labeled with CellTrace Violet (Thermo) as described previously [32]. Briefly, sorted T cells were co-cultured with WT mitomycin-treated and T-depleted splenocytes as APCs, at a 1:5 T cell:APC ratio, in IMDM. Anti-CD3 (0.01, 0.1 or 1 ug/ml) and anti-CD28 (3 ug/ml) were added, and the cells were cultured for 3 days. Cultures were restimulated with 1 ug/ml anti-CD3 + 3 ug/ml anti-CD28, and blocked with 1:1000 dilution of GolgiStop (BD Biosciences), for 4 h. Cells were stained with LIVE/DEAD Fixable Aqua Dead Cell Stain, fixed in 4% PFA, permeabilized, washed and stained in PBS + 0.1% BSA + 0.5% Triton X-100.

### Immunization and ELISA

Sigma Adjuvant System (Sigma Aldrich) was reconstituted in 1 ml PBS, warmed to 37C, vortexed, and 10 ul of the suspension was mixed with 100 ug NP_16_-ova (Biosearch) for intraperitoneal injection. Sheep red blood cells (Colorado Serum Company) were counted on a ViCell (Beckman Coulter), then 2.5×10^8^ cells were diluted in PBS for intraperitoneal injection. Spleens and serum were analyzed 7–8 days post-immunization. Total serum IgG ELISA was performed as described previously [33]. For antigen-specific antibody titers, ELISA plates were coated with 5 ug/ml of NP_20_-BSA (Biosearch), and the assay was performed as described previously [34]. Arbitrary units of antigen-specific antibodies were calculated according to reference serum from NP_16_-OVA hyper-immunized mice.

All relevant data are within the paper and its Supporting Information files.

## Results

### Generation of *Slamf1/Slamf5/Slamf6* triple knockout mice using CRISPR-mediated genomic engineering

**Fig 1:**
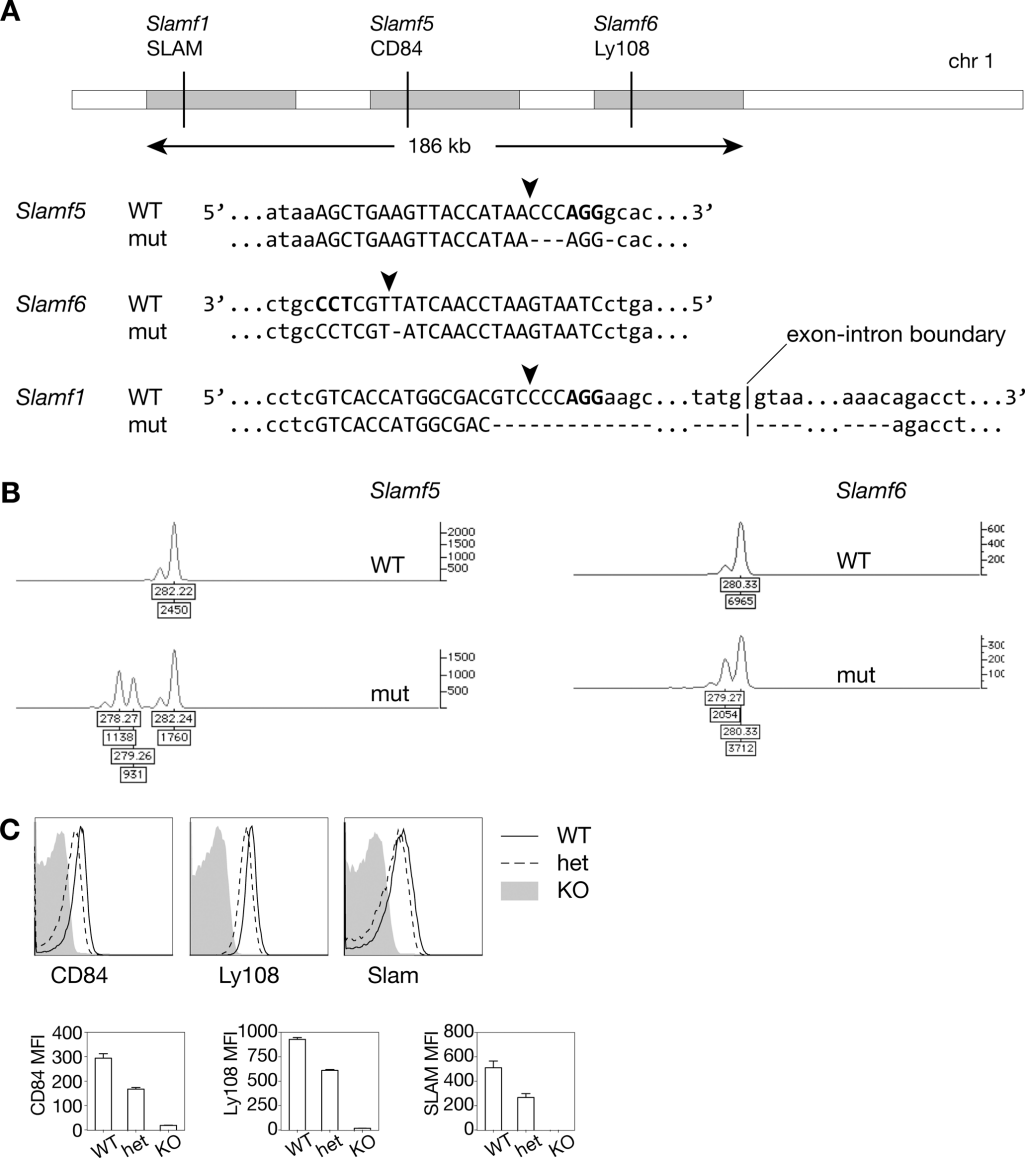
Generation of a *Slamf1*^-/-^*Slamf5*^-/-^*Slamf6*^-/-^ triple knockout mouse line on a pure C57BL6 background by microinjecting *S*. *pyogenes* Cas9 mRNA and sgRNA into fertilized eggs. (A) The *Slamf1*, *Slamf5*, *Slamf6* locus is located on mouse chromosome 1. Vertical lines represent guide sequence target sites. Genomic DNA sequences of the targeted loci are shown for both a WT control and the TKO mouse. Guide sequences are in upper case, PAM sequences are bolded, predicted Cas9 cut sites are marked by arrows, and deletions are denoted by dashes. **(B)** Fluorescent PCR chromatograms showing sizes of target loci PCR amplicons for *Slamf5* and *Slamf6* in a WT control and the TKO founder. Each peak represents a differently sized mutant allele amplicon. **(C)** Representative CD84, Ly108, and SLAM expression on peripheral blood B cells (B220^+^) from TKO mice and littermates. Median fluorescence intensities of CD84, Ly108, and SLAM, n = 3–6 mice/genotype, error bars show s.e.m.

To simultaneously disrupt the three *Slamf* genes of interest, we selected three guide RNA sequences, one targeting each gene, based on off-targeting scores from crispr.mit.edu (Fig 1A, S1 Table). In the first injection, we also included a donor oligo for each gene, containing a stop codon and a restriction enzyme site (S1 Table). Founders were screened for integration of the donor oligo by PCR of the targeted region and restriction enzyme digest. However, oligo integration was not found in any of the founders, indicating that homology-directed repair was inefficient in this injection. To rapidly screen for small mutations in the targeted genes, we used a high-throughput fluorescent PCR assay that can detect indels at single base pair resolution [30]. In this assay, the targeted genomic region was amplified by two gene-specific primers, as well as a third fluorophore-tagged universal primer (S2 Table), to generate amplicons of 250–400 bp length, which were measured on a capillary sequencer in comparison to a DNA size standard (Fig 1B). This method is particularly useful for mosaic samples, since both the amplicon size and relative abundance of each mutant allele can be detected; the height of the mutant peak reflects the percent of the DNA carrying the mutation. Thus, larger peaks are more prevalent in the founder, and more likely to be transmitted to the offspring. Of the 36 founders, 11 had mutations in *Slamf1*, three in *Slamf6*, and two in *Slamf5* (S3 Table). Of the mice harboring mutations in *Slamf6*, all had mutations in *Slamf1*. The two mice harboring mutations in *Slamf5* also carried mutations in the other two *Slam* family members (S3 Table). In several samples, there were more than two mutant peaks for a single gene, each with a distinct height, suggestive of mosaicism (Fig 1B), which has been previously reported in CRISPR-generated mice, likely as the result of Cas9 nuclease activity occurring after the first zygotic division [35]. Therefore, not all of the mutations detected in the founder are necessarily transmitted to the offspring. For some founders in this and subsequent injections, single mutant peaks were detected in the absence of WT peaks (S1 Table), indicating that both alleles were mutated.

In a second injection, we chose two new guides each for *Slamf5* and *Slamf6*, and one for *Slamf1* (S2 Table and S1 Fig). We selected guides that started with one or two G’s, based on work on CRISPR-based transgenic zebrafish that showed the importance of starting sgRNAs with GG, both for efficient in vitro transcription from the T7 promoter, as well as mutagenesis efficiency in vivo [36]. The five new sgRNAs and the *Slamf1* sgRNA from the first injection were combined with Cas9 mRNA for the second injection. As measured by fluorescent PCR, higher rates (9–11 pups, 39–48%) of small indels were obtained for all three genes, compared to the first injection (S1 Table). Nearly all of the mutant mice had mutations in more than one of the three genes (S1 Table). Consistent with previous reports [26], the majority of mutations were small deletions that are predicted to cause frame-shift mutations and premature stop codons (S1 Table). Moreover, the sizes of some deletions were compatible with deletions occurring between the two guides for a given gene. Collectively, these results confirmed that CRISPR-mediated mutagenesis is a rapid and efficient method to generate transgenic mice with multiple genetic modifications simultaneously.

One founder from the first injection had 3 and 4 bp deletions in *Slamf5*, a 1 bp deletion in *Slamf6*, and an 8 bp deletion in *Slamf1*, as detected by fluorescent PCR (Fig 1B). This founder was crossed to C57Bl/6 mice. Although none of the F1 progeny appeared to have out of frame mutations in all three alleles by our fluorescent PCR assay, a fraction of the F1 progeny carried both the 4 bp deletion in *Slamf5* and the 1 bp insertion in *Slamf6*. These F1 mice were interbred with each other to generate mice homozygous for these two mutations. To confirm the loss of expression of these SLAMF members, we analyzed peripheral blood lymphocytes from the F2 progeny mice by flow cytometry. Surprisingly, evaluation of the expression of SLAM, CD84, and Ly108 revealed that homozygous mice had lost expression of all three receptors (Fig 1C). Further analyses revealed that these mice also carried a 370 bp deletion in *Slamf1* that spanned the remainder of the exon downstream of the cut site, as well as a portion of the subsequent intron (Fig 1A). Because the fluorescent PCR assay has an amplicon size limit of 400 bp, and one of the primer sequences for *Slamf1* was within the deleted region, our original PCR assay only amplified the WT allele and not this allele. Thus, this mutation was originally missed in the founder and F1 mice. Expression of adjacent genes including those encoding Ly9 *(Slamf3)* and CD48 (*Slamf2*) were not affected in these mice (S2 Fig). We therefore chose to use these triple knockout mice for *Slamf1*, *Slamf5*, and *Slamf6* (TKO) for subsequent analyses.

### Slamf156 TKO mice exhibit normal development of conventional lymphocytes

**Fig 2:**
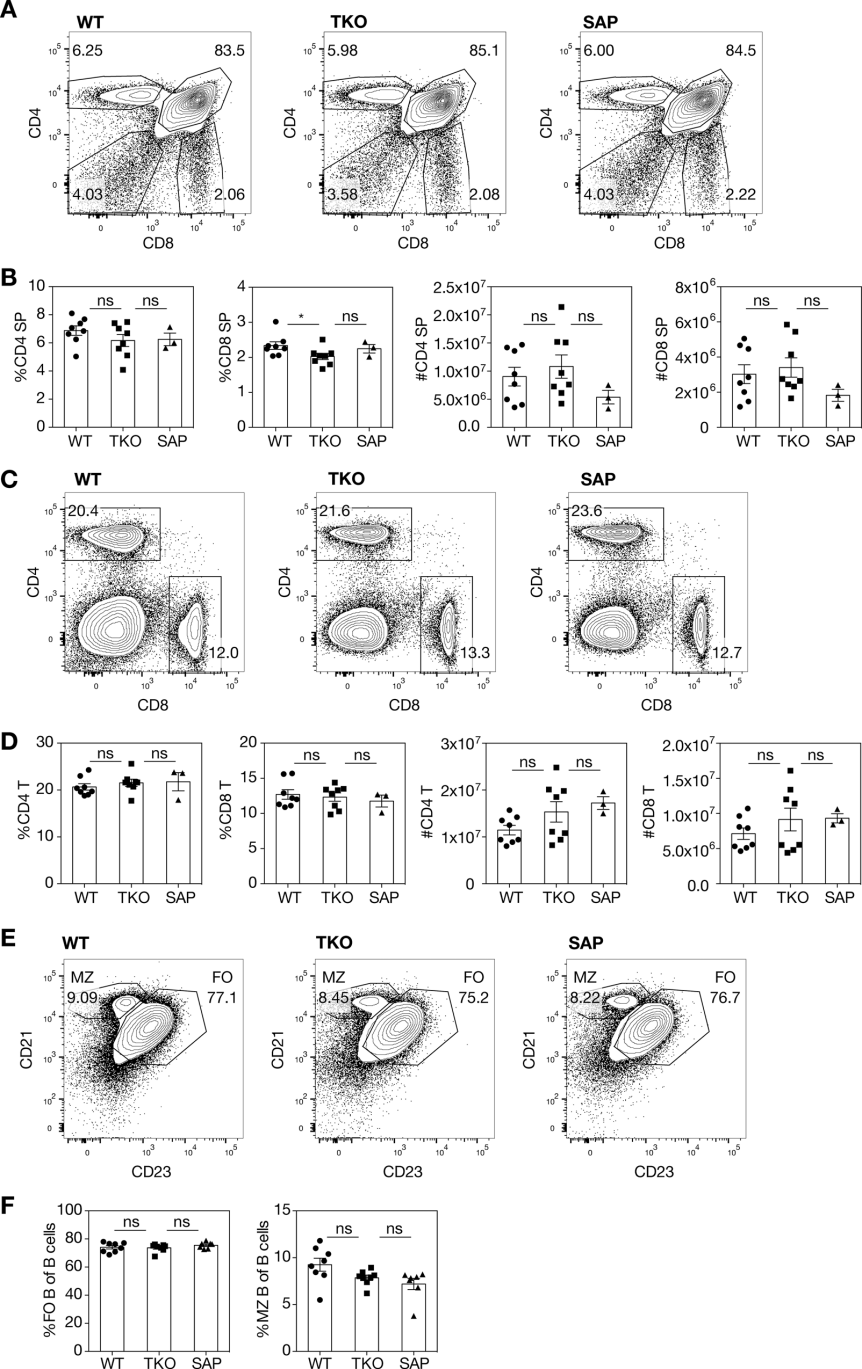
Normal development of thymic conventional αβ T cells, peripheral CD4^+^ and CD8^+^ T cells, and splenic B cells in TKO mice. **(A)** Representative flow cytometry plots and **(B)** quantitation of single positive thymocytes. Data were pooled from 2 independent experiments, n = 3–8 mice/genotype. (**C)** Representative flow cytometry plots and **(D)** quantitation of splenic CD4^+^ and CD8^+^ T cells. Data were pooled from 2 independent experiments, n = 3–8 mice/genotype. (**E**) Representative flow plots and **(F)** quantitation of follicular (FO) and marginal zone (MZ) splenic B cells. Data were pooled from 2 independent experiments, n = 7–8 mice/genotype. Error bars show s.e.m., group means were compared by *t*-test, ns = not significant, *p<0.05.

Previous studies established that SAP-deficiency does not affect the development of conventional αβ T cells or B cells in mice [27, 37]. Consistent with these data, flow cytometry analyses revealed that TKO mice had grossly normal thymic development, with populations of single- and double-positive thymocytes that were comparable to those of wild-type (WT) mice (Fig 2A and 2B). Similar percentages of innate-like CD8^+^CD44^+^CD122^+^ cells were also found in the TKO thymus as in WT mice (S3 Fig). In the spleens of TKO mice, the percentages and numbers of CD4^+^ and CD8^+^ T cells were also identical to those in WT and SAP-deficient mice (Fig 2C and 2D). The spleen also contained normal marginal zone and follicular B cell populations in the TKO mice (Fig 2E and 2F). Thus, development of conventional lymphocytes was grossly intact in the absence of these three SLAM family members.

### iNKT cell development is defective in Slamf156 TKO mice

**Fig 3:**
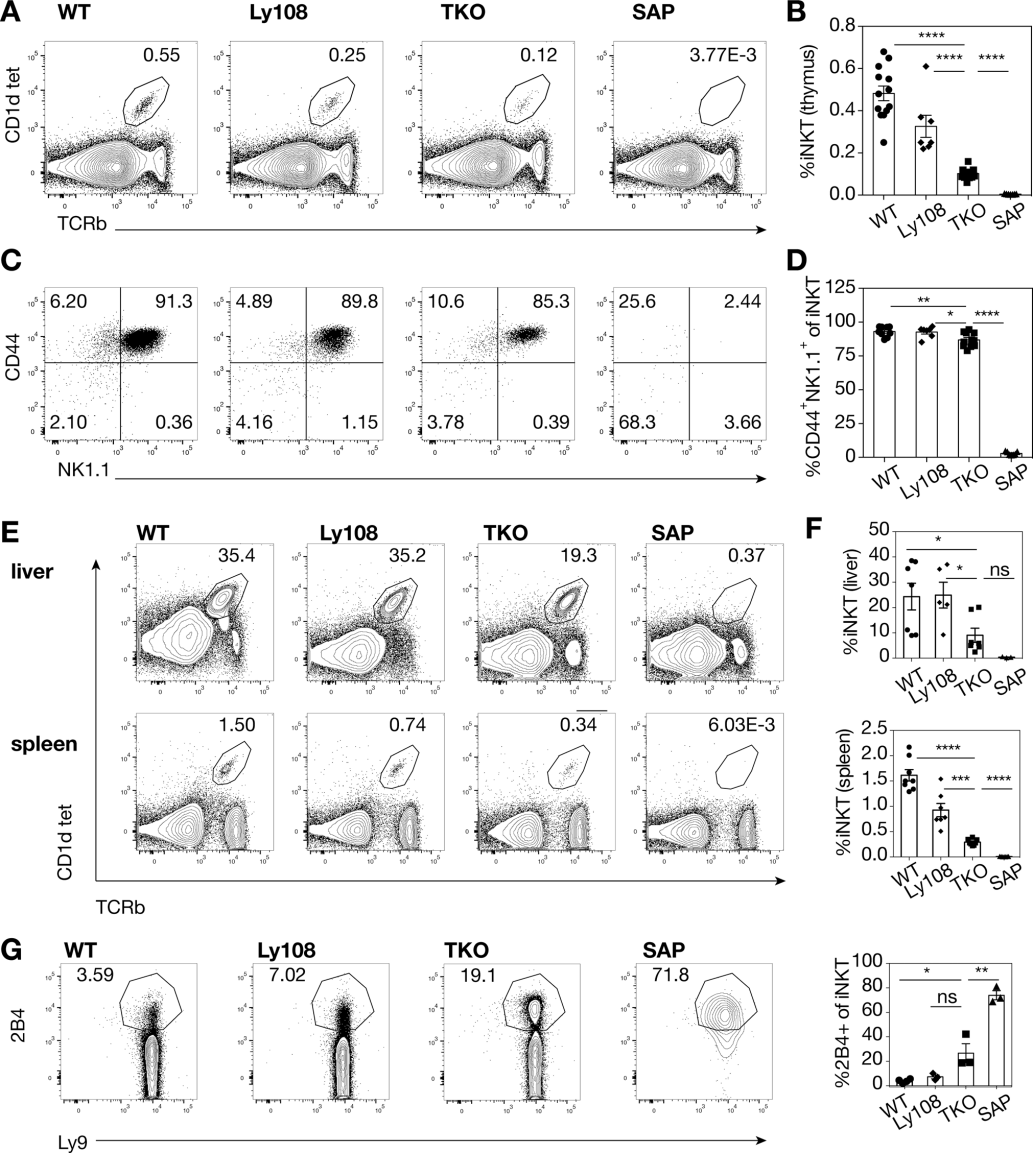
Impaired iNKT cell development in TKO mice. **(A)** Representative flow cytometry plots and **(B)** quantitation of thymic iNKT cells. Data were pooled from 4 independent experiments, n = 7–15 mice/genotype. **(C)** Representative flow cytometry plots and **(D)** quantitation of mature CD44^+^NK1.1^+^ thymic iNKT cells. Data were pooled from 3 independent experiments, n = 7–11 mice/genotype. **(E)** Representative flow cytometry plots and **(F)** quantitation of hepatic and splenic iNKT cells. Data were pooled from 2 independent experiments, n = 3–7 mice/genotype for liver, n = 7–8 mice/genotype for spleen. **(G)** Representative flow cytometry plots and quantitation of 2B4 expression on liver iNKT cells. Representative data from 1 of 2 independent experiments is shown, n = 3–4 mice/genotype. Error bars show s.e.m., group means were compared by *t*-test, ns = not significant, *p<0.05, **p<0.01, ***p<0.001, ****p<0.0001.

One of the most dramatic phenotypes associated with SAP-deficiency is a lack of iNKT cells [5–7]. While no single SLAM family member appears to be responsible for this phenotype, mice deficient in Ly108 exhibit the greatest phenotype, showing about 50% reductions in iNKT cells [25, 38]. However, mixed bone marrow chimeras that force selection on both SLAM and Ly108 suggest that both these two receptors participate in iNKT development [25]. Consistent with potential roles for both receptors, the TKO mice exhibited decreased iNKT cell development with even lower frequencies than seen in *Slamf6*^-/-^ mice (Fig 3A and 3B). Nonetheless, the TKO iNKT cells that did develop were able to progress to mature CD44^+^NK1.1^+^ cells (Fig 3C and 3D).

As expected from their low thymic output, iNKT cells were also reduced in the liver and spleen of TKO mice (Fig 3E and 3F). Furthermore, TKO iNKT cells showed altered expression of other SLAM family members, having an expanded 2B4-high population, particularly in the liver (Fig 3G and 3H). Notably, although these phenotypes were less dramatic than those seen in *Sh2d1a*^-/-^ mice, again, they were more severe than seen in *Slamf6*^-/-^ mice, supporting evidence for SLAM family redundancy in iNKT cell development.

### Slamf156 TKO CD4 T cells proliferate and produce IL-4 similarly to WT cells in vitro

**Fig 4:**
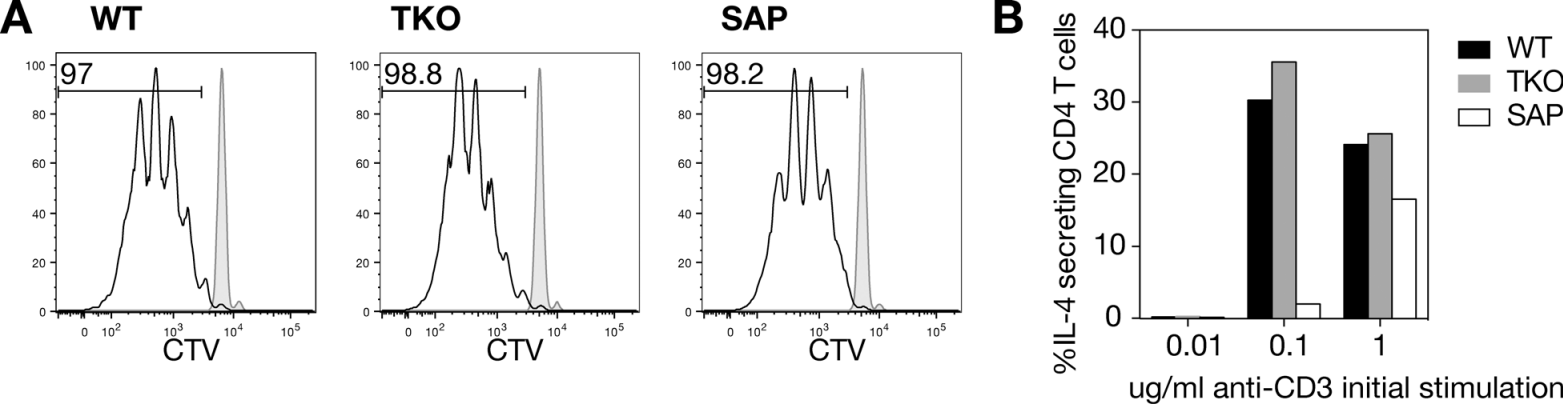
TKO CD4 T cells proliferated and secreted IL-4 similarly to WT cells in response to TCR activation in vitro. Naïve CD4 T cells were sorted and cultured with WT antigen presenting cells, in the presence of anti-CD3 and anti-CD28. **(A)** Cell proliferation was measured by CellTrace Violet after 3 days of co-culture with WT APCs, in the presence of 0.1 ug/ml anti-CD3 and 3 ug/ml anti-CD28. The grey filled histogram represents control cells cultured with APCs but without anti-CD3 and anti-CD28, for each genotype. **(B)** IL-4 production was measured by restimulating and intracellular staining of cells after 3 days of co-culture with WT APCs, in the presence of 3 ug/ml anti-CD28 and varying concentrations of anti-CD3. Representative data from 1 of 2 independent experiments is shown.

To assess the functionality of TKO CD4 T cells, we sorted naïve CD4 T cells from WT, TKO and SAP mice, labeled them with CellTrace Violet, co-cultured them with WT antigen presenting cells, and added anti-CD3 and anti-CD28 stimulation. Previous studies have shown that purified SAP CD4 T cells proliferate normally after anti-CD3 and anti-CD28 stimulation [22]. Consistent with this, after three days, similar T cell proliferation was observed for all three genotypes (Fig 4A).

In the absence of exogenous cytokine signals, SAP CD4 T cells are poor producers of IL-4 in response to TCR stimulation in vitro [22, 37]. To evaluate whether TKO CD4 T cells also have IL-4 production defects, we activated naïve CD4 cells without polarizing cytokines, restimulated them with anti-CD3 and anti-CD28, and then stained for intracellular production of IL-4. In this setting, TKO and WT CD4 T cells produced similar levels of IL-4, regardless of the initial anti-CD3 stimulation (Fig 4B). Thus, the TKO CD4 T cells have normal proliferation and IL-4 production in response to TCR stimulation.

### Slamf156 TKO mice have modestly decreased responses to protein immunization

**Fig 5:**
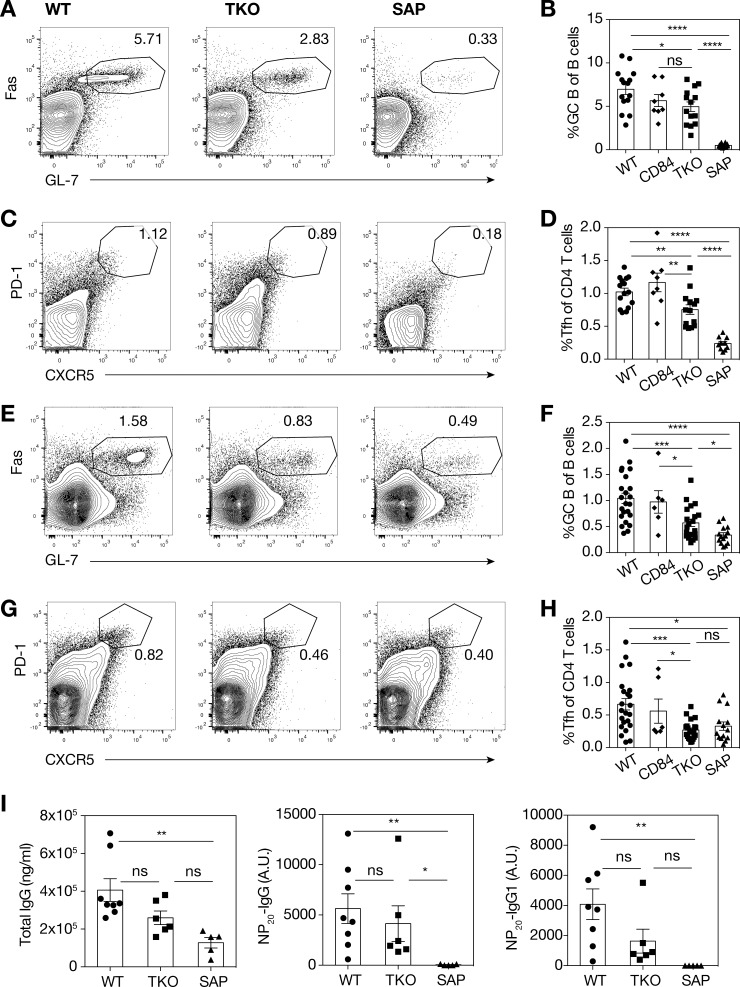
TKO mice show mildly reduced responses to immunization with sheep RBC or NP-ova. (A) Representative flow cytometry plots and **(B)** quantitation of GC B cells, **(C)** representative flow cytometry plots and **(D)** quantitation of Tfh cells from spleen, day 8 post-immunization with sheep RBCs. Data were pooled from 3 independent experiments, n = 10–15 mice/group. **(E)** Representative flow cytometry plots and (**F)** quantitation of GC B cells, **(G)** representative flow cytometry plots and **(H)** quantitation of Tfh cells from spleen, day 7 post-immunization I.P. with NP-ova and Sigma Adjuvant System. Data were pooled from 4 independent experiments, n = 14–23 mice/group. GC B cells were gated on live CD19^+^B220^+^Fas^+^GL-7^+^ cells, Tfh cells were gated on live CD4^+^B220^-^CXCR5^hi^PD-1^hi^ cells. **(I)** Serum antibody titer ELISA, day 7 post-immunization with NP-ova and Sigma Adjuvant System. Concentration of antibodies for individual mice (NP-specific concentrations are relative to a pooled positive control), A.U. = arbitrary units. Data is representative of 1 of 2 independent experiments, n = 5–8 mice/group. Error bars show s.e.m., group means for (B, D, F, H) were compared by *t*-test, group means for (I) were compared by nonparametric Kruskal-Wallis test, followed by Dunn’s post hoc test, ns = not significant, *p<0.05, **p<0.01, ***p<0.001, ****p<0.0001.

SAP-deficient mice exhibit dramatic defects in humoral immune responses, due to the requirement of SAP for maintenance of stable Tfh:B cell interactions [8]. To assess whether SLAM, CD84 and Ly108 play a coordinated role in responses to protein immunization, we challenged mice with the T-dependent antigen, sheep red blood cells (SRBCs). Consistent with previous studies [39], *Sh2d1a*^-/-^ mice had very poor germinal center B cell formation (Fig 5A and 5B), associated with reduced GC (CD4^+^CXCR5^hi^PD-1^hi^) Tfh cells (Fig 5C and 5D). Evaluation of responses of TKO mice revealed lower frequencies of Fas^+^GL-7^+^ germinal center B cells than seen in WT mice (Fig 5A and 5B), suggesting a mild defect in germinal center responses, similar to that seen with CD84-deficient mice in the same experiment. However, while cumulatively statistically significant, the extent of this defect was variable, and only statistically significant in two of three individual experiments. CXCR5^hi^PD1^hi^ Tfh cell frequencies were also slightly reduced compared to WT; again, the magnitude of the reduction was variable (Fig 5C and 5D). Similarly, following intraperitoneal immunization with another T-dependent antigen, NP-conjugated ovalbumin (NP-ova), TKO mice also showed GC B and Tfh defects that reached statistical significance when data were pooled (Fig 5E–5H), but in only two out of four individual experiments. Consistent with this, serum antibody titers measured by ELISA showed that TKO mice had antigen-specific IgG and IgG1 responses that were intermediate between those of WT and SAP-deficient mice (Fig 5I). Nonetheless, we did not detect significant differences in plasma cell percentages between WT and TKO mice (S4 Fig). Thus, in both immunization settings, the TKO mice showed decreased responses, but these were less severe than those of *Sh2d1a*^-/-^ mice.

## Discussion

The importance of the SLAM family of receptors is underscored by the phenotypes associated with mutations affecting SAP, a small adaptor molecule that binds the SLAM family members and engages the Src family kinases, Fyn and Lck. Mutations affecting SAP lead to the human genetic disease X-linked Lymphoproliferative Syndrome Type 1. However, which and how SLAM family members contribute to the distinct phenotypes associated with SAP-deficiency have remained unclear. Here, we used Cas9/CRISPR-based mutagenesis to mutate the three SLAM family members that are highest expressed in GC B and Tfh cells, SLAM (*Slamf1*), CD84 (*Slamf5*), and Ly108 (*Slamf6*), all of which are homophilic and mediate cell-cell interactions [19]. Our results suggest that these receptors have some functional redundancy in positive signaling, particularly in iNKT development. However, the relatively mild phenotypes of these mice further support a major role for inhibitory signaling from SLAM family members as contributing to phenotypes observed in the absence of SAP.

Data suggest SLAM family members can relay both positive and negative signaling [1, 40]. Given the strong phenotype of SAP-deficient mice, many studies focused on positive signaling roles downstream of SLAMF members, providing evidence that SAP recruits Src family kinases and other signaling molecules [41, 42]. However, early reports also noted that the binding site for SAP resembled a binding site for phosphatases [17], suggesting that this site could function as an “immunotyrosine switch motif” (ITSM), allowing both positive and negative signaling [43]. Data from NK cells in particular, provided evidence for inhibitory signaling from SLAM family members, notably 2B4, in the absence of SAP and the SAP-related adaptor, EAT2 [14, 44]. More recently, several groups, including ours, have provided evidence that SLAM family members also act as inhibitory receptors in Tfh:B cell interactions [15] and in the regulation of killing of B cell targets by cytotoxic T lymphocytes [10–12]. These studies demonstrate that the SLAM family members Ly108 (and 2B4 on CD8 cells) recruit the SHP-1 phosphatase and other negative signaling molecules in the absence of SAP, leading to a strong inhibition of TCR signaling and altered synapse formation upon interactions with B cells and probably other hematopoietic cells that express high levels of the homotypic SLAM family members [10, 15, 16]. Remarkably, blocking or mutating Ly108 could completely rescue GC formation, iNKT cell development and CD8 cytolysis of B cells in the absence of SAP, implicating negative signaling from SLAM family members as a major cause of these phenotypes associated with SAP-deficiency [10–12, 15]. These observations suggested the relatively mild GC phenotypes of individual SLAM family knockouts could reflect their primary and strong function as inhibitory receptors in the absence of SAP. However, it was still possible that functional redundancy between these receptors had masked their positive roles in pathways controlling these phenotypes.

Although mutation of SLAM family members has revealed intriguing functions in innate cells including neutrophils and macrophages [23], phenotypes in T cells have been relatively mild. SAP-deficient mice have dramatic defects in GC formation [4], yet, individual SLAM family members do not show such phenotypes [15, 19, 21]. Both deficiency and overexpression studies in culture have pointed to a role for SLAM in promoting IL-4 expression [22, 23, 45], potentially via a PKC-θ-mediated pathway. This has been supported by data showing defective IL-4 expression from T cells of SLAM-deficient mice in vivo [21, 46] and other data implicating a role for SLAM in regulating a conserved noncoding sequence in the *Il4* gene in Tfh cells [47, 48]. Nonetheless, we did not see evidence for decreased IL-4 production in CD4 cells from our TKO mice. CD84-deficient mice can show decreased responses to protein immunization. However, again we observed mild intermediate defects in response to protein and SRBC immunization that were similar or only slightly reduced compared to those seen in the absence of CD84, and which did not approach the defects associated with SAP-deficiency. Thus, although we cannot rule out a requirement for other SLAM family members for GC responses, the defects observed in SAP-deficient mice and humans with XLP may well result from inhibitory signaling in the absence of SAP, as suggested by the rescue of GC formation in *Slamf6*^-/-^*Sh2d1a*^-/-^ mice.

Perhaps the strongest data supporting positive signaling roles for SLAM family members comes from iNKT cell development. Ly108-deficiency reduces iNKT cells by 50% [25, 38]. Moreover, both Ly108 and SLAM have been implicated as having roles in iNKT development in experiments using mixed bone marrow chimeras [25]. Supporting this view, our data demonstrate that the TKO of SLAM, CD84 and Ly108 worsens the phenotype of Ly108-deficient mice. It therefore is of note, that while Ly108-deficiency largely rescued iNKT cell development in SAP-deficient mice, it only rescued cell numbers to the level of iNKT cells found in Ly108-deficient mice, again supporting that Ly108 signaling plays a positive role in iNKT cell development [15]. As that iNKT cells are positively selected on other DP thymocytes, and require selection on hematopoietic cells, the hematopoietically-expressed SLAM family members are likely candidates for supplying positive signals required for their development. Positive roles in iNKT cell development are also consistent with the observation that Fyn-deficient animals have reduced iNKT development—i.e. the phenotype is partially phenocopied by mutations affecting a downstream positive regulator of SLAM family signaling [6, 7].

In this work, the multiplex nature of CRISPR-mediated mutagenesis offered a significant advantage towards the study of combinatorial knockout of multiple linked genes. Rather than using distinct constructs for successive mutations, CRISPR allows the simple combination of guide RNAs to target different genes, thereby vastly accelerating the rate at which the transgenic mice can be obtained. While the TKO mouse was obtained in an injection with relatively low mutation frequencies, optimized injection conditions and use of two targeting guides per gene significantly increased our mutation rates (>80% per gene in more recent experiments, S1 Table). Furthermore, the 370 bp deletion in the *Slamf1* gene of the TKO mice shows that large deletions can occur even with a single guide RNA cut, which may not be detected by the fluorescent PCR screening assay. Therefore, the true mutagenesis rates may have been higher, if there were large deletions surrounding single guide RNA cut sites, or larger, even intergenic, deletions spanning two cut sites [49].

An additional advantage of CRISPR-based genetic engineering is that it can be applied directly in fertilized eggs of specific desired strains. In the case of the SLAM family members, polymorphisms between strains can affect susceptibility to autoimmunity [50]. Similarly, polymorphisms in humans have been linked to autoimmunity [51]. Previous studies of *Slamf1*^-/-^ [21] and *Slamf6*^-/-^ [19] mice that were backcrossed to C57Bl/6 mice have used lupus-prone (129 x B6) Sle1b mice as WT controls, due to residual 129-derived sequences around the SLAM loci, which were mutated in 129 ES cells. To bypass these potentially confounding effects, we performed our mutagenesis directly in oocytes derived from C57BL/6 (B6) crosses. The microinjection of B6 eggs further allowed direct breeding of homozygous offspring in a pure B6 background.

Recently, Terhorst and colleagues also generated a *Slamf156* triple knockout mouse on a B6 background, using conventional transgenic ES cell technology in two steps to delete the entire region [24]. Consistent with our findings, the Terhorst TKO model also had grossly normal thymic lymphocyte development, but significantly reduced numbers of iNKT cells [52]. Peripheral lymphocyte development was also mostly normal in their TKO mouse, although they observed increased numbers of marginal zone B cells and increased CD8^+^CD122^+^ innate-like cells [24], which we did not see in our experiments.

After NP-ova immunization, the Terhorst TKO mouse had increased NP-specific IgG levels [24], despite normal GC B cells and Tfh cell numbers. They attributed the higher antibody response to increased numbers of plasma cells, which we again did not observe. Mixed adoptive transfer experiments with mutant and WT B and T cells further suggested the increased antibody response in their *Slamf156* model was primarily B cell-intrinsic. Interestingly, antibody responses were higher in their TKO model than in a *Slamf16* double knockout they generated, yet their *Slamf5*^-/-^ model showed no phenotype, in contrast to previous work from our group [19]. Furthermore, data from the Crotty laboratory on another CRISPR-derived triple knockout of these SLAM family members suggest that there is no GC phenotype in mice lacking these three receptors, despite clear defects in NKT cell development (S. Crotty, personal communication). We have also observed that the TKO showed even weaker defects when immunized under different conditions (i.e. in draining lymph nodes after high levels of NP-ova injected subcutaneously, B.H. unpublished observations). These observations suggest that mutations affecting SLAMF members may exhibit relatively modest GC phenotypes that may differ depending on the presence or absence of the other family members, the type of mutation generated (as in the Terhorst mutants containing deletions spanning multiple genes), or other factors such as the specific assays or microbiota that may vary. Nonetheless, the relatively modest GC phenotypes support the idea that the severity of humoral immune defects in XLP1 patients and in SAP-deficient mice may result primarily from inhibitory signaling from these receptors. How combined mutations of other *Slam* family members affect other pathways, such as cytolytic activity or cell survival will be of interest. Furthermore, why dual signaling has evolved for these receptors in regulating interactions between lymphocytes remains an important and intriguing question.

## Supporting Information

S1 Fig Schematic of guide sequences used in the micro-injections. For each gene, the targeted exon is represented by a grey bar. The guide sequences used in the first injection (one per gene) are represented by dashed arrows. Those used in the second injection (two per gene) are represented by solid black arrows, with the distance between each pair of predicted cut sites labeled Δx. The direction of the arrow indicates whether the guide sequence is on the sense strand (forward) or antisense strand (reverse).
(PDF)

S2 Fig Comparable CD48 and Ly9 expression in WT and TKO mice. Representative flow cytometry plots of CD48 and Ly9 in spleen of WT and TKO mice. B cells were gated on live B220^+^ cells, and T cells were gated on live CD4^+^ plus live CD8^+^ cells. Data are representative of 2 independent experiments, n = 4 mice/genotype.
(PDF)

S3 Fig Normal frequencies of innate-like CD8^+^ T cells in thymus of TKO mice. Representative flow cytometry plots of innate-like CD8^+^CD44^+^CD122^+^ T cells, gated on CD8^+^CD4^-^ single positive cells. Data are representative of 2 independent experiments, n = 4 mice/genotype.
(PDF)

S4 Fig Comparable frequencies of plasma cells following protein immunization of WT and TKO mice. Quantitation of plasma cells in the spleen, 7 days post-immunization I.P. with NP-ova and Sigma Adjuvant System. Plasma cells were gated on live CD19^med^CD138^+^ cells. Data were pooled from 2 independent experiments, n = 12–13 mice/genotype. Error bars show s.e.m., group means were compared by *t*-test, ns = not significant.
(PDF)

S1 Table Mutations in founders derived from simultaneous injection of sgRNAs targeting *Slamf1/5/6*, as measured by fluorescent PCR. Each subsequent injection had higher mutation frequencies in each of the three genes, higher frequencies of founder mice with mutations in more than one gene, and more homozygous mutations (mutant peaks without a wt peak).
(PDF)

S2 Table Sequences of the 20-nucleotide guides, the concentrations used in the microinjections, the guide sequence oligos used for cloning into the T7 sgRNA vector (Transposagen), and the donor oligos. Overhangs are in bold, restriction sites are highlighted in grey.
(PDF)

S3 Table Sequences of primers for genotyping SLAMF knockout mice. M13 and PIGTAIL sequences are in bold. Annealing temperature of 60C was used for all reactions.
(DOCX)
