# Supplementary material for: CRISPR-Mediated Triple Knockout of SLAMF1, SLAMF5 and SLAMF6 Supports Positive Signaling Roles in NKT Cell Development
Source: PLoS One. 2016 Jun 3;11(6):e0156072. doi: 10.1371/journal.pone.0156072 (PMC4892526; doi:10.1371/journal.pone.0156072)
Supplement: S1 Fig — For each gene, the targeted exon is represented by a grey bar. The guide sequences used in the first injection (one per gene) are represented by dashed arrows. Those used in the second injection (two per gene) are represented by solid black arrows, with the distance between each pair of predicted cut sites labeled Δx. The direction of the arrow indicates whether the guide sequence is on the sense strand (forward) or antisense strand (reverse). (PDF) [file pone.0156072.s001.pdf]

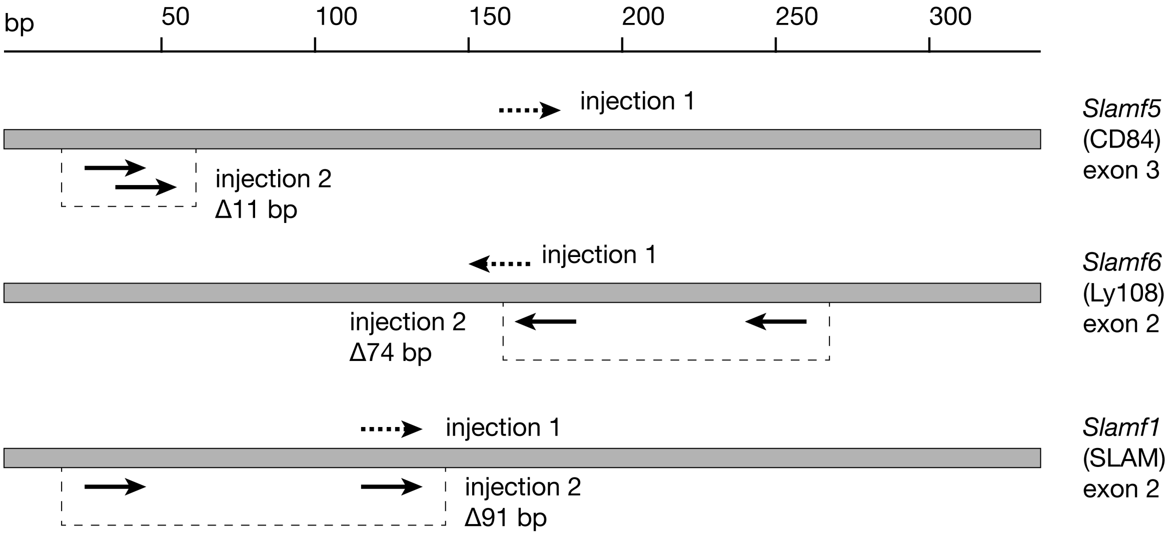

S1 Figure. Schematic of guide sequences used in the micro-injections. For each gene, the targeted exon is represented by a grey bar. The guide sequences used in the first injection (one per gene) are represented by dashed arrows. Those used in the second injection (two per gene) are represented by solid black arrows, with the distance between each pair of predicted cut sites labeled Δx. The direction of the arrow indicates whether the guide sequence is on the sense strand (forward) or antisense strand (reverse).
