# Supplementary material for: CRISPR-Mediated Triple Knockout of SLAMF1, SLAMF5 and SLAMF6 Supports Positive Signaling Roles in NKT Cell Development
Source: PLoS One. 2016 Jun 3;11(6):e0156072. doi: 10.1371/journal.pone.0156072 (PMC4892526; doi:10.1371/journal.pone.0156072)
Supplement: S2 Fig — Representative flow cytometry plots of CD48 and Ly9 in spleen of WT and TKO mice. B cells were gated on live B220+ cells, and T cells were gated on live CD4+ plus live CD8+ cells. Data are representative of 2 independent experiments, n = 4 mice/genotype. (PDF) [file pone.0156072.s002.pdf]

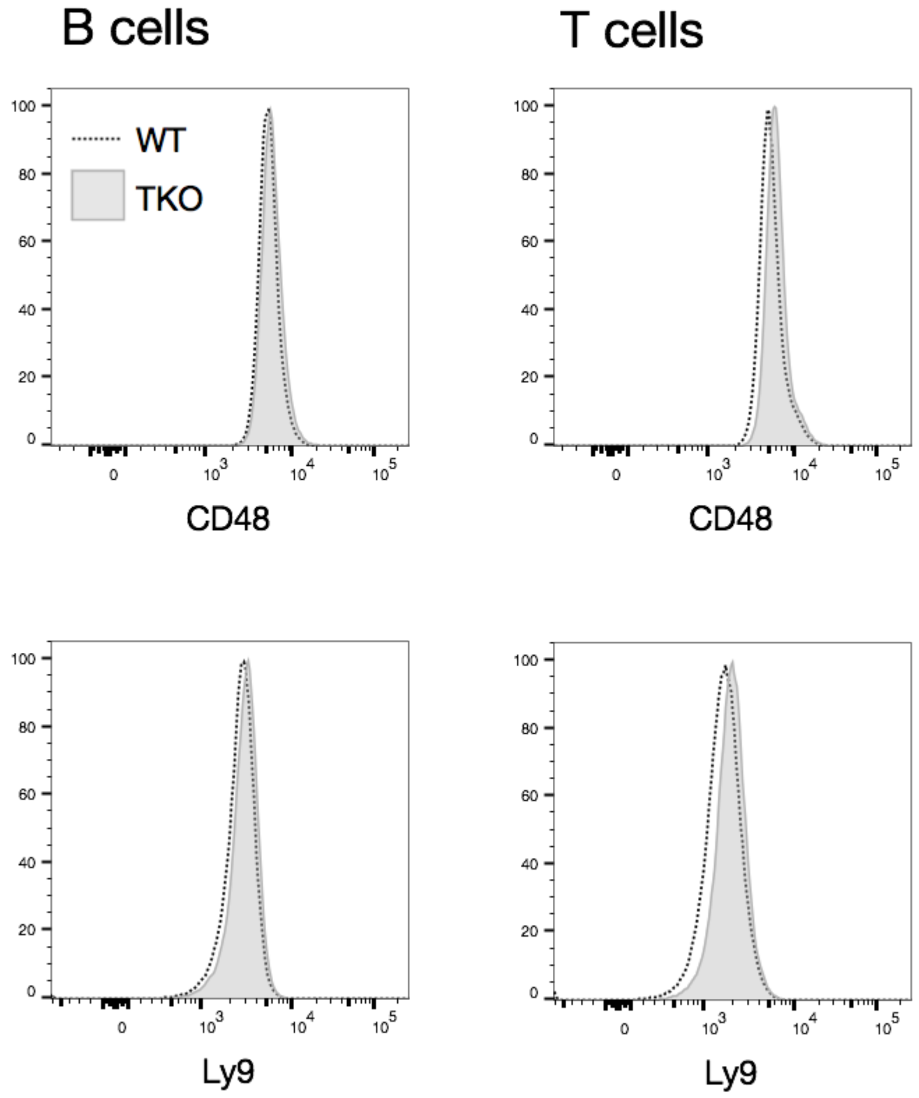

S2 Figure. Comparable CD48 and Ly9 expression in WT and TKO mice. Representative flow cytometry plots of CD48 and Ly9 in spleen of WT and TKO mice. B cells were gated on live B220$^+$ cells, and T cells were gated on live CD4$^+$ plus live CD8$^+$ cells. Data are representative of 2 independent experiments, n=4 mice/genotype.
