# Supplementary material for: CRISPR-Mediated Triple Knockout of SLAMF1, SLAMF5 and SLAMF6 Supports Positive Signaling Roles in NKT Cell Development
Source: PLoS One. 2016 Jun 3;11(6):e0156072. doi: 10.1371/journal.pone.0156072 (PMC4892526; doi:10.1371/journal.pone.0156072)
Supplement: S3 Fig — Representative flow cytometry plots of innate-like CD8+CD44+CD122+ T cells, gated on CD8+CD4- single positive cells. Data are representative of 2 independent experiments, n = 4 mice/genotype. (PDF) [file pone.0156072.s003.pdf]

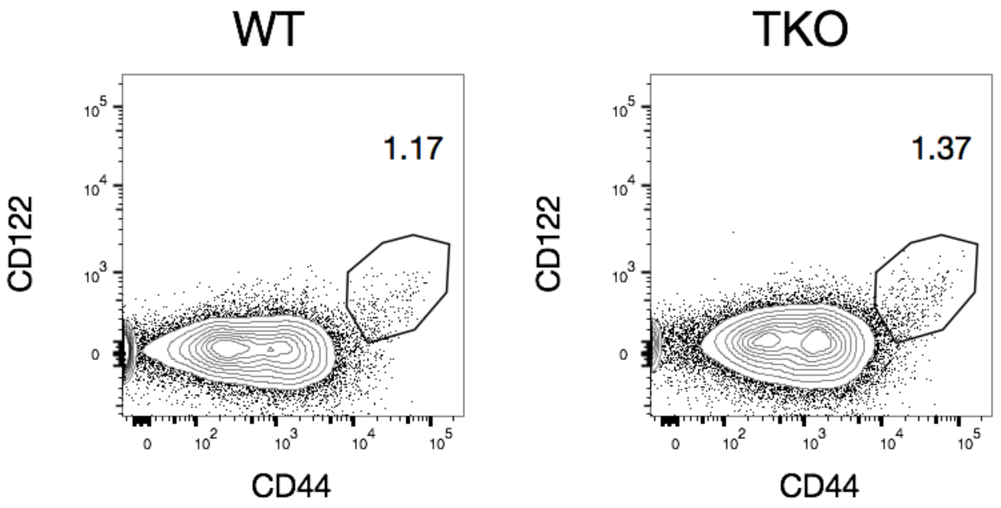

S3 Figure. Normal frequencies of innate-like CD8^{+} T cells in thymus of TKO mice. Representative flow cytometry plots of innate-like CD8^{+}CD44^{+}CD122^{+} T cells, gated on CD8^{+}CD4^{-} single positive cells. Data are representative of 2 independent experiments, n=4 mice/genotype.
