# Supplementary material for: CRISPR-Mediated Triple Knockout of SLAMF1, SLAMF5 and SLAMF6 Supports Positive Signaling Roles in NKT Cell Development
Source: PLoS One. 2016 Jun 3;11(6):e0156072. doi: 10.1371/journal.pone.0156072 (PMC4892526; doi:10.1371/journal.pone.0156072)
Supplement: S4 Fig — Quantitation of plasma cells in the spleen, 7 days post-immunization I.P. with NP-ova and Sigma Adjuvant System. Plasma cells were gated on live CD19medCD138+ cells. Data were pooled from 2 independent experiments, n = 12–13 mice/genotype. Error bars show s.e.m., group means were compared by t-test, ns = not significant. (PDF) [file pone.0156072.s004.pdf]

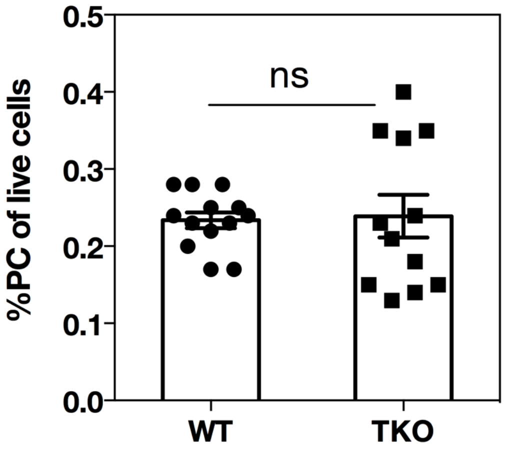

S4 Figure. Comparable frequencies of plasma cells following protein immunization of WT and TKO mice. Quantitation of plasma cells in the spleen, 7 days post-immunization I.P. with NP-ova and Sigma Adjuvant System. Plasma cells were gated on live $CD19^{med}CD138^{+}$ cells. Data were pooled from 2 independent experiments, n=12-13 mice/genotype. Error bars show s.e.m., group means were compared by *t*-test, ns = not significant.
