# Supplementary material for: CRISPR-Mediated Triple Knockout of SLAMF1, SLAMF5 and SLAMF6 Supports Positive Signaling Roles in NKT Cell Development
Source: PLoS One. 2016 Jun 3;11(6):e0156072. doi: 10.1371/journal.pone.0156072 (PMC4892526; doi:10.1371/journal.pone.0156072)
Supplement: S1 Table — Each subsequent injection had higher mutation frequencies in each of the three genes, higher frequencies of founder mice with mutations in more than one gene, and more homozygous mutations (mutant peaks without a wt peak). (PDF) [file pone.0156072.s005.pdf]

**Founder**	***Slamf5***	***Slamf6***	***Slamf1***
**Injection 1, total 36 pups**			
Total mutated pups	2	3	11
%mutated pups	5.6%	8.3%	31%
A1			-15, +99, wt
A3	-3	+1	+1, wt
A4			-11, -4, -3, wt
A5	-4, -3, wt	-1, wt	-8, wt
A6			-15, -3, +81, wt
A8		+1, wt	-15, +12, wt
C5			-14, -2, wt
C6			+4, wt
C9			-15, wt
C11			+2, wt
D2			-16, wt
**Injection 2, total 23 pups**			
Total mutated pups	9	11	9
%mutated pups	39%	48%	39%
A0		-4, -27, wt	
A1			+39, +80, wt
A4	-3, -9, -2	-4, +1, -90, -9, -3, +2	
A5	-4, wt	-89	-6, -3, -1, +1
B1	-25, +9, wt	+2, -90, -27, wt	-12, -11, -8
B2	-3, -12, -2	-56, -12, +1, -20, -17, -7, -4	-17, -8, -16, -1, wt
B3	-9, wt	-10, -74, -11	-8, wt
B4	+9	-22, -27, wt	-12, -11
B5			-24, -2, -23, wt
C3		-4, -74, -3	+62, -27
D0	-9, -8, wt	-74, wt	
D1	-37	-78, wt	
D3	-6, -22, -9	-4, +1, -12, -7, -3, +2	-6, -3, -1, wt
**Injection 3a, total 11 pups**			
Total mutated pups	6	8	N/A
%mutated pups	55%	73%	N/A
D1		-10, +2	
D2			
D3	+1, wt	-75, -10, -4	
D4		-4, -3, wt	

D5	-4, +1	-4, -1	
D6			
D7	+1, wt	-11, -4, -2, +1	
D8	-10, wt	-22, -19	
D9	-6, wt	-10	
D10			
D11	+1, wt	-13, -11, wt	
**Injection 3b, total 9 pups**			
Total mutated pups	8	8	N/A
%mutated pups	89%	89%	N/A
E2	-10, -9, +1	-8, -3, wt	
E3	-10, -3, -1	-15, -8, -1, wt	
E4	+1	-90, -15, -8, -3, wt	
E5	-3, +1, +2	-5, -1, wt	
E6	-1	-15, wt	
E7	-10, +1	-7	
E8	-10	-2, wt	
E9			
E10	-1	-13, -5, wt	

S1 Table. Mutations in founders derived from simultaneous injection of sgRNAs targeting *Slamf1/5/6*, as measured by fluorescent PCR. Each subsequent injection had higher mutation frequencies in each of the three genes, higher frequencies of founder mice with mutations in more than one gene, and more homozygous mutations (mutant peaks without a wt peak).
