# Supplementary material for: CRISPR-Mediated Triple Knockout of SLAMF1, SLAMF5 and SLAMF6 Supports Positive Signaling Roles in NKT Cell Development
Source: PLoS One. 2016 Jun 3;11(6):e0156072. doi: 10.1371/journal.pone.0156072 (PMC4892526; doi:10.1371/journal.pone.0156072)
Supplement: S2 Table — Overhangs are in bold, restriction sites are highlighted in grey. (PDF) [file pone.0156072.s006.pdf]

Target	Guide sequence	Final Concentration (ng/ul)	Cloning oligos
**Injection 1**			
Slamf5	AGCTGAAGTTACCATAACCC	2.5	**TAGG**AGCTGAAGTTACCATAACCC<br>**AAAC**GGGTTATGGTAACTTCAGCT
Slamf6	GATTACTTAGGTTGATAACG	2.5	**TAGG**GATTACTTAGGTTGATAACG<br>**AAAC**CGTTATCAACCTAAGTAATC
Slamf1	GTCACCATGGCGACGTCCCC	2.5	**TAGG**GTCACCATGGCGACGTCCCC<br>**AAAC**GGGGACGTCGCCATGGTGAC
SpCas9		5	
Donor oligo	See below	10	
Slamf5 donor oligo	GAACCAAAGAAAATTGACAACATTGCCTGGACTTCTCAATCATCTGTTGCTTTTATAAAACCAGG<br>AGTCAATAAAGCTGAAGTTACCATATAAGCTTGCACTTATAAAGGACGAATAGAAATCATAGATC<br>AGAAGTATGACCTGGTCATTAGAGACCTGAGGATGGAAGATGCAGGAACTTAC		
Slamf6 donor oligo	AGGAGAGTCTGCAGTTCTTCCTCTAAAGCTTCCTGCAGGGAAGATAGCCAATATCATCATCTGGA<br>ATTATGAATGGGAAGCGTCACAAGTCACTTGAGGATCCTATCAACCTAAGTAATCCTGAAAGTCC<br>ACAAATCATGAACACTGATGTAAAGAAGAGACTGAACATCACCCAGTCCTACTC		
Slamf1 donor oligo	CACGTGGCTGCCCCTGACGAATGAACATCAGATAAATAAGAGCGTGAACAAAAGTGTCCGCATCC<br>TCGTCACCATGGCGACGTCCTAAGCTTAAGCAAATCCAACAAGAAAATTGTGTCTTTTGATCTCT<br>CTAAAGGGAGCTATCCAGATCACCTGGAGGATGGCTACCACTTTCAATCA		
**Injection 2**			
Slamf5	GGTAATGAATGGGATTCTTG	5	**TAGG**TAATGAATGGGATTCTTG<br>**AAAC**CAAGAATCCCATTCATTA
	GCAGACCCGGTGGTAATGAA	5	**TAGG**CAGACCCGGTGGTAATGAA<br>**AAAC**TTCATTACCACCGGGTCTG
Slamf6	GGTAAGGTTGCTGATTTGCA	5	**TAGG**TAAGGTTGCTGATTTGCA<br>**AAAC**TGCAAATCAGCAACCTTA
	GTGGACTTTCAGGATTACTT	5	**TAGG**TGGACTTTCAGGATTACTT<br>**AAAC**AAGTAATCCTGAAAGTCCA
Slamf1	GATTCTCCAGAAGCTGGGAC	5	**TAGG**ATTCTCCAGAAGCTGGGAC<br>**AAAC**GTCCCAGCTTCTGGAGAAT
	GTCACCATGGCGACGTCCCC	5	**TAGG**GTCACCATGGCGACGTCCCC<br>**AAAC**GGGGACGTCGCCATGGTGAC
SpCas9		50	
**Injection 3a**			
Slamf5	GCAGACCCGGTGGTAATGAA	10	**TAGG**CAGACCCGGTGGTAATGAA<br>**AAAC**TTCATTACCACCGGGTCTG
Slamf6	GTGGACTTTCAGGATTACTT	10	**TAGG**TGGACTTTCAGGATTACTT<br>**AAAC**AAGTAATCCTGAAAGTCCA
SpCas9		50	
**Injection 3b**			
Slamf5	GGTAATGAATGGGATTCTTG	10	**TAGG**TAATGAATGGGATTCTTG

			**AAAC**CAAGAATCCCATTCATTA
Slamf6	GGTAAGGTTGCTGATTTGCA	10	**TAGG**TAAGGTTGCTGATTTGCA<br>**AAAC**TGCAAATCAGCAACCTTA
SpCas9		50	

S2 Table. Sequences of the 20-nucleotide guides, the concentrations used in the microinjections, the guide sequence oligos used for cloning into the T7 sgRNA vector (Transposagen), and the donor oligos. Overhangs are in bold, restriction sites are highlighted in grey.
