# Supplementary material for: CRISPR-Mediated Triple Knockout of SLAMF1, SLAMF5 and SLAMF6 Supports Positive Signaling Roles in NKT Cell Development
Source: PLoS One. 2016 Jun 3;11(6):e0156072. doi: 10.1371/journal.pone.0156072 (PMC4892526; doi:10.1371/journal.pone.0156072)
Supplement: S3 Table — M13 and PIGTAIL sequences are in bold. Annealing temperature of 60C was used for all reactions. (DOCX) [file pone.0156072.s007.docx]

| **Founder** | ***Slamf5*** | ***Slamf6*** | ***Slamf1*** |
| --- | --- | --- | --- |
| **Injection 1, total 36 pups** | | | |
| Total mutated pups | 2 | 3 | 11 |
| %mutated pups | 5.6% | 8.3% | 31% |
| A1 |  |  | -15, +99, wt |
| A3 | -3 | +1 | +1, wt |
| A4 |  |  | -11, -4, -3, wt |
| A5 | -4, -3, wt | -1, wt | -8, wt |
| A6 |  |  | -15, -3, +81, wt |
| A8 |  | +1, wt | -15, +12, wt |
| C5 |  |  | -14, -2, wt |
| C6 |  |  | +4, wt |
| C9 |  |  | -15, wt |
| C11 |  |  | +2, wt |
| D2 |  |  | -16, wt |
| **Injection 2, total 23 pups** | | | |
| Total mutated pups | 9 | 11 | 9 |
| %mutated pups | 39% | 48% | 39% |
| A0 |  | -4, -27, wt |  |
| A1 |  |  | +39, +80, wt |
| A4 | -3, -9, -2 | -4, +1, -90, -9, -3, +2 |  |
| A5 | -4, wt | -89 | -6, -3, -1, +1 |
| B1 | -25, +9, wt | +2, -90, -27, wt | -12, -11, -8 |
| B2 | -3, -12, -2 | -56, -12, +1, -20, -17, -7, -4 | -17, -8, -16, -1, wt |
| B3 | -9, wt | -10, -74, -11 | -8, wt |
| B4 | +9 | -22, -27, wt | -12, -11 |
| B5 |  |  | -24, -2, -23, wt |
| C3 |  | -4, -74, -3 | +62, -27 |
| D0 | -9, -8, wt | -74, wt |  |
| D1 | -37 | -78, wt |  |
| D3 | -6, -22, -9 | -4, +1, -12, -7, -3, +2 | -6, -3, -1, wt |

### **S3 Table.** **Mutations in founders derived from simultaneous injection of sgRNAs targeting *Slamf1/5/6*, as measured by fluorescent PCR.** The second injection had higher mutation frequencies in all three genes, and higher frequencies of founder mice with mutations in more than one gene.
